# Higher thymocyte selection-associated high mobility group box (TOX) expression predicts poor prognosis in patients with ovarian cancer

**DOI:** 10.1186/s12885-022-10336-6

**Published:** 2022-11-25

**Authors:** Sai Li, Sifu Yang, Yupeng Hong

**Affiliations:** 1 Department of gynecologic oncology, Women’s Hospital, Zhejiang University school of medicine, Hangzhou, China; 2 Cancer Center, Department of Medical Oncology, Zhejiang Provincial People’s Hospital, Affiliated People’s Hospital, Hangzhou Medical College, Hangzhou, Zhejiang China

**Keywords:** Ovarian cancer, TOX, CD8 + T cell, Prognosis

## Abstract

**Background:**

Ovarian cancer is one of the most lethal gynecologic malignancies with a dismal prognosis that poses a serious threat to human health, highlighting the need for more knowledge about what is required for identifying some biomarkers for early diagnosis, prediction of prognosis and disease monitoring. TOX, a critical transcription factor related to the development of malignancies that contributing to lymphocytes not just T cells, had been proved prognostic value in some spectrum of cancers. Here, we aimed to study the prognostic role of TOX in ovarian cancer.

**Results:**

We found that TOX was not only expressed in CD8 T cells but also tumor cells. TOX expression score was higher in ovarian cancer tissues and correlated with survival status. Survival analysis revealed that ovarian cancer patients with high TOX expression score generally shorter overall survival and disease-free survival times. Univariate and Multivariate Cox demonstrated that TOX expression score could be used as an independent prognostic factor for patients with ovarian cancer.

**Conclusion:**

TOX expression in ovarian cancer could be a promising tool for predict overall survival of ovarian cancer patients.

## Introduction

Ovarian cancer is the one of the most lethal gynecologic malignancies [1]. High-grade serous ovarian cancer (HGSC) is the most prevalent histological subtype of epithelial ovarian cancer that usually presents diffuse carcinogenesis in the advanced stage with only 20-30% 5-year survival rate [2, 3]. The main reason of its high mortality is due to its insidious symptoms at early stage. Over 70% of ovarian cancer patients were diagnosed as FIGO stage III or IV at their initial presentation [4]. Despite significant improvements have been made in surgical managements and systemic therapeutic treatments, high recurrence rate and chemotherapy resistant rate are the main reason for miserable survival rate [4, 5]. Therefore, it is critically important to identify some biomarkers for early diagnosis, prediction of prognosis and disease monitoring.

TOX (thymocyte selection-associated high mobility group box) is nuclear DNA-binding factor and a member of the high-mobility group box superfamily that is thought to bind DNA in a sequence-independent manner but structure-dependent manner [6]. TOX comprises four subfamily members (TOX1-4, TOX1 is also known as TOX) [6] and is a critical transcription factor related to the development of malignancies that contributing to T cells and other lymphocytes [7–9]. Recently, as a critical regulator of T lymphocyte differentiation, TOX was found to be involved in CD8 + T cell exhaustion and epigenetically reprogram CD8 + T cells to drive exhaustion [10–14]. Collectively, TOX can act as a key inducer of canonical features of T cell exhaustion and an initiator of the T exhaustion cell specific epigenetic program [15]. The tumor microenvironment (TME) is composed of diverse cell types including immune cells, stromal cells, vascular networks and some tissue-specific cell types [16]. Of note, it has been well recognized that TME can have a profound impact on the cancer progression and therapeutic outcome [17–19]. Thus, the expression levels of TOX maybe reflect the function of CD8 T cell in the tumor microenvironment and thus can predict the prognosis of cancer patients. For example, some studies illustrated that TOX was positively correlated with larger tumor size, lower differentiation, advanced TNM stage and predicting exhaustion and low infiltration of tumor infiltrating CD8 + T cells in tumor microenvironment, highlighting a potential biomarker for cancer immunotherapy efficacy [10, 11, 20–22]. Nevertheless, the correlations between TOX and prognosis in different types of cancer including ovarian cancer remain elusive.

In this study, we comprehensively investigated the expression characteristics and prognostic value of the TOX in ovarian cancer. The Survival analysis and a Cox regression model were employed to identify the correlation between TOX expression and ovarian cancer patients’ survival rate. The results found that TOX was a potential prognosis-related biomarker in ovarian cancer and provided novel direction to understand the interactions between TOX expression, tumor infiltration, and T cells exhaustion.

## Results

### Localization and expression of TOX in ovarian cancer tissues

**Fig. 1:**
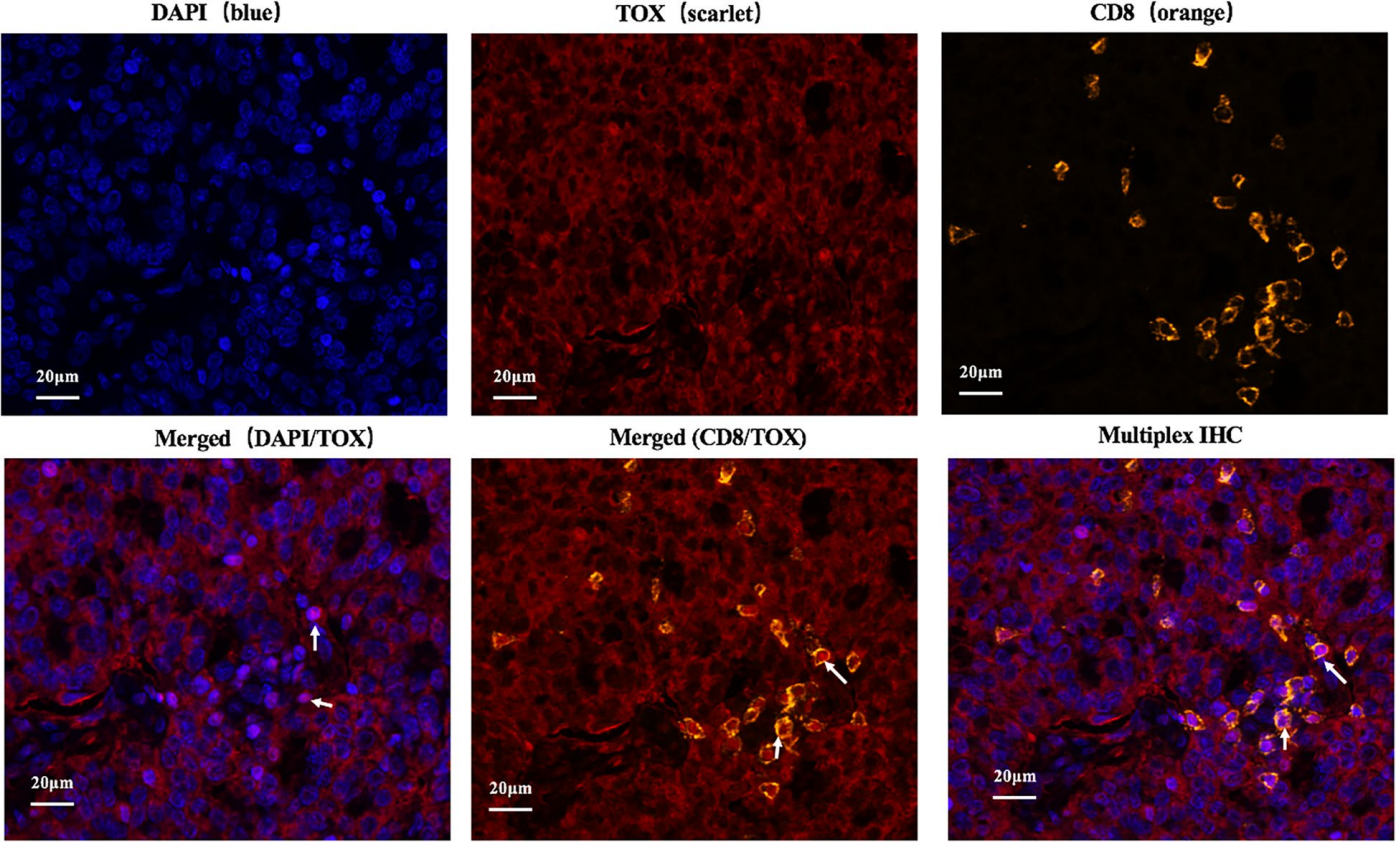
Representative immunofluorescent staining of ovarian cancer from a patient with high grade serous ovarian cancer. The sample was stained for TOX (scarlet), CD8 (orange) and DAPI (blue)

**Fig. 2:**
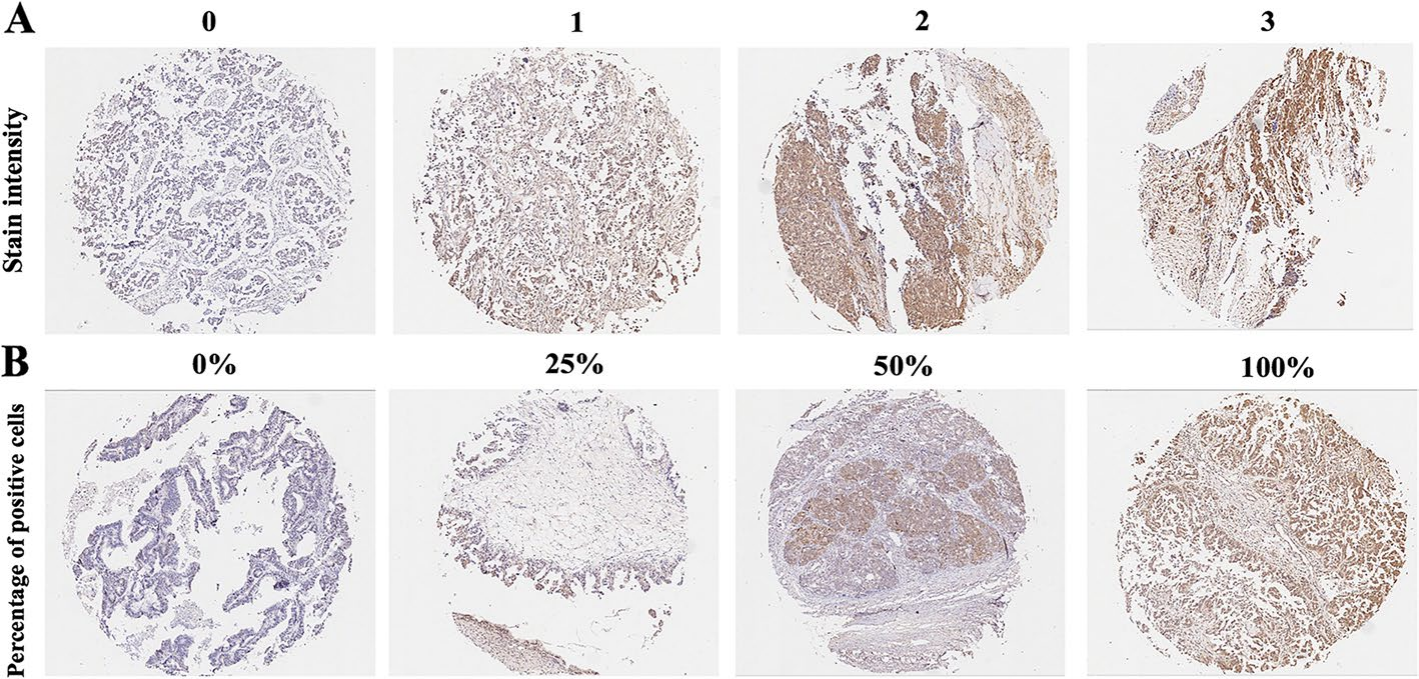
Representative images of TOX staining in ovarian cancer tissue.**A** Stain intensity was evaluated with a 4-grading system: 0 = negative, 1 = weak, 2 = moderate and 3 = strong. **B **The percentage of positive cells were scored as follows: 0 for no cell stained, 1 for 1 − 25% of cells stained, 2 for 26–50% of cells stained, 3 for 51–75% of cells stained and 4 for more than 75% of cells stained

**Fig. 3:**
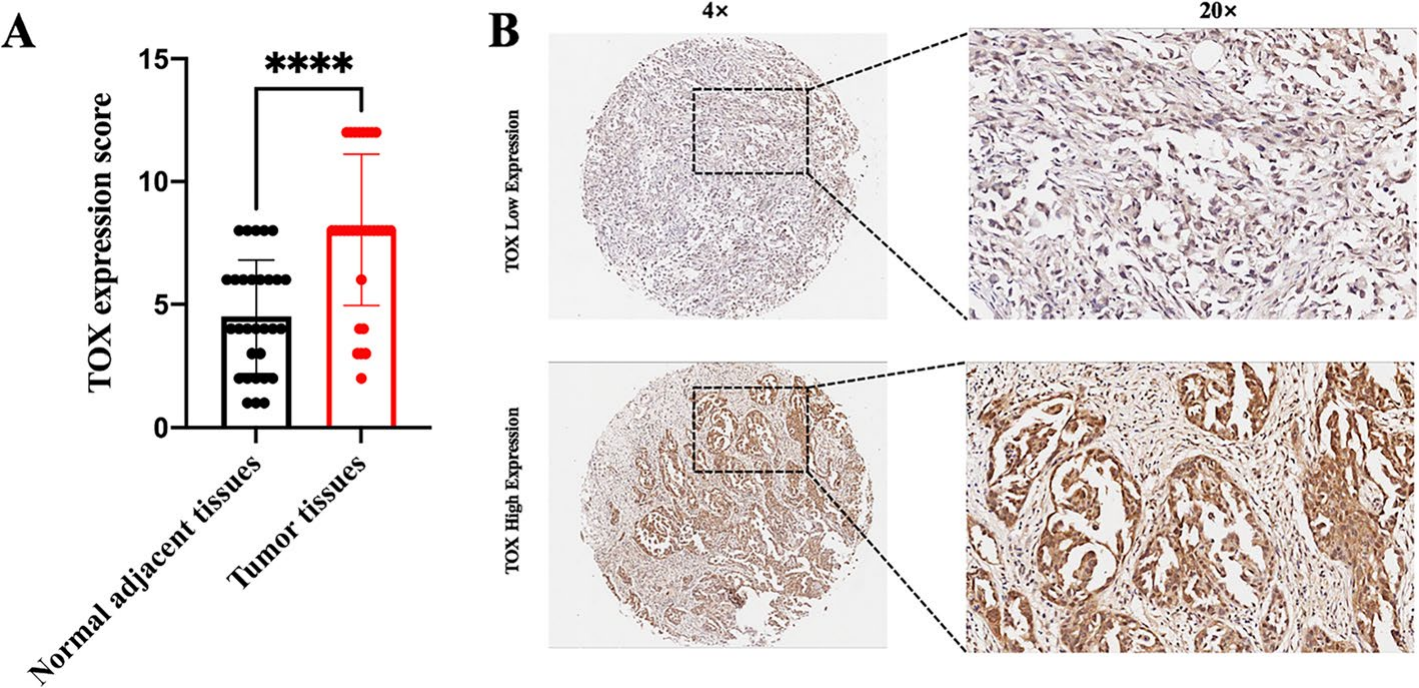
TOX expression scores are higher in tumor tissues that in normal adjacent tissues.**A** TOX expression scores are compared between tumor tissues and normal adjacent tissues (Statistical significance was determined using the Mann-Whitney U test.). **B** low TOX expression score and high TOX expression score stain pattern in ovarian cancer

We sought to understand the localization of TOX in the ovarian cancer milieu. Multiple immunofluorescence staining showed that TOX was not only expressed in the tumor cells but also in CD8 + T cells (Fig. 1). TOX expression in surgically resected specimens form ovarian cancer were assessed by IHC(Immunohistochemistry) staining (Fig. 2). As shown in Fig. 3, TOX expression was elevated in tumor tissues compared to normal adjacent tissues. Variations in TOX expression were observed across tumor tissues. The average expression of TOX in tumor tissues was much higher than in normal adjacent tissues.

### Association of TOX expression with clinicopathological parameters in patients with ovarian cancer

The correlations between TOX and clinicopathological features in tissue specimens from 116 patients with ovarian cancer were illustrated in Table 1. The patients were categorized as TOX high and TOX low according to the expression of TOX. TOX expression in tumors was demonstrated to be positively correlated with tumor status (tumor differentiation grade(p^**^<0.01); pathological assessment of the primary tumor (T stage, p^**^<0.01); regional lymph nodes (N stage, p^***^<0.001); distant metastasis (M stage, p^***^<0.001) and clinical stage (TNM stage, p^***^<0.001)). Moreover, it was shown that the expressions of Ki67 and PD-L1 were positively correlated with TOX (p^**^<0.01). Whereas TOX expression was not associated with the age of patients and expression of EGFR in tumors. The results showed that Spearman’s correlation coefficients for TOX and Ki67, EGFR and PD-L1 were 0.321 (*p* < 0.001), 0.155 (*p* = 0.098) and 0.125 (*p* = 0.180) respectively. It demonstrated a positive correlation of TOX expression score with Ki67, but no correlations with EGFR and PD-L1.

**Table 1:** Correlation between TOX expression and clinicopathological characteristics

	Variables	TOX expression		Total	*p* value
		Low	High		
Age (year)					0.345
	≤ 50	30	29	59	
	> 50	24	33	57	
Grade					0.0085^**^
	I-II	13	6	19	
	III	32	65	97	
T stage					0.003^**^
	I-II	23	11	34	
	III	31	51	82	
N stage					< 0.001^***^
	N0	47	35	82	
	N1	7	27	34	
M stage					< 0.001^***^
	M0	50	37	87	
	M1	4	25	29	
TNM stage					< 0.001^***^
	I-II	23	11	34	
	III	27	26	53	
	IV	4	25	29	
KI67					0.002^**^
	Low	33	20	53	
	High	21	42	63	
EGFR					0.690
	Low	29	31	60	
	High	25	31	56	
PDL-1					0.021^*^
	Low	23	14	37	
	High	31	48	79	

Statistically significant: p^*^<0.05, p^**^<0.01, p^***^<0.001, *p* values were analyzed by chi-square test, adjusted chi-square test, or Fisher’s exact test

TNM stage: American Joint Committee on Cancer’s cancer staging, 8th edition

### High TOX expression is associated with a poor OS of patients with ovarian cancer

**Fig. 4:**
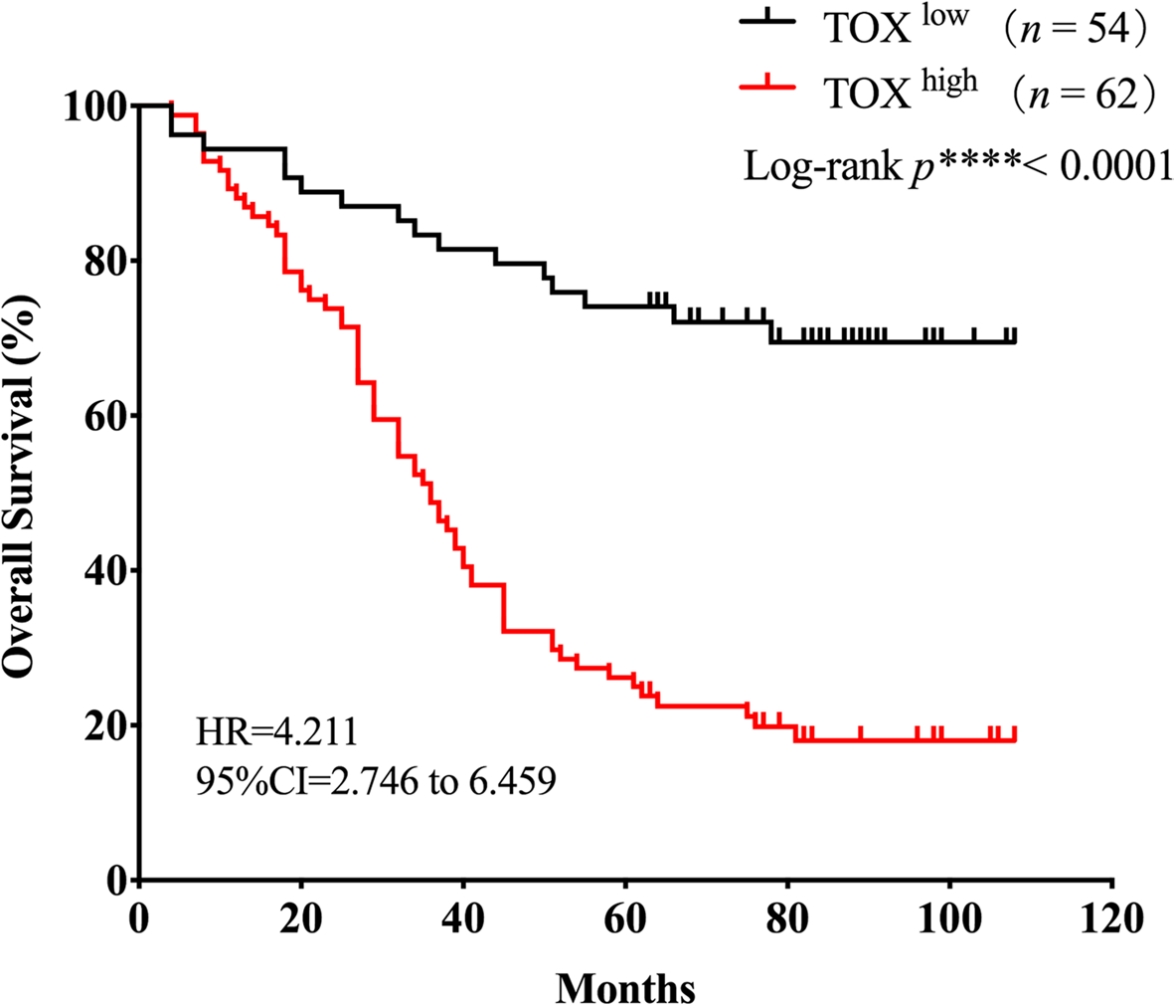
Kaplan-Meier survival curves for overall survival of patients with ovarian cancer according to the TOX expression scores. Overall survival curves for patients according to the low(*n* = 54) and high(*n *= 62) expression levels of TOX in ovarian cancer tissues (log-rank test and *p* values are shown)

The Kaplan-Meier curves indicated that patients with high TOX expression were remarkably correlated with short OS (Overall survival) than those with low TOX expression (Log-rank p^****^< 0.0001, Hazard Ratio = 4.211,95%CI = 2.746 to 6.459, Fig. 4). The median OS of patients with high TOX expression was 36 months, whereas that of patients with low TOX expression was still not reached (Fig. 4).

As shown in Table 2, a univariate Cox regression analysis revealed that TOX expression was an independent predictor of short OS (Hazard Ratio = 4.062, 95%CI = 2.297–7.182, p^***^<0.001). As expected, other independent parameters including T stage stage, M stage and TNM stage were also identified as independent predictors of OS. In addition, a multivariate Cox model also suggested that TOX expression was an independent risk factor for OS in patients with ovarian cancer (Hazard Ratio = 2.255, 95%CI = 1.199–4.241, p^*^<0.05). The multivariate Cox model also demonstrated that T stage and M stage were both independent risk factors for OS in ovarian cancer, but not N stage. This finding suggested that TOX expression in ovarian cancer could be a promising tool for predict OS of ovarian cancer patients.

**Table 2:** Univariate and Multivariate Cox analysis of overall survival in ovarian cancer

Variables	Univariate analysis			Multivariate analysis		
	HR	95%CI	*p* value	HR	95%CI	*p* value
TOX expression	4.062	2.297–7.182	< 0.001^***^	2.255	1.199–4.241	0.012^*^
Age	1.384	0.848–2.258	0.194			
Grade	1.512	0.745–3.071	0.252			
T stage	8.329	3.122–22.224	< 0.001^***^	4.925	1.843–13.157	0.001^***^
N stage	5.106	3.097–8.418	< 0.001^***^	1.419	0.712–2.829	0.320
M stage	6.120	3.612–10.367	< 0.001^***^	2.151	1.061–4.359	0.034^*^
TNM stage	4.480	2.982–6.729	< 0.001^***^			
KI67	1.455	0.882–2.400	0.142			
EGFR	0.838	0.514–1.366	0.479			
PDL-1	1.737	0.988–3.056	0.055			

Statistically significant (p^*^ < 0.05, p^**^ < 0.01, p^***^ < 0.001)

### High TOX expression correlates with poor DFS in patients with ovarian cancer

**Fig. 5:**
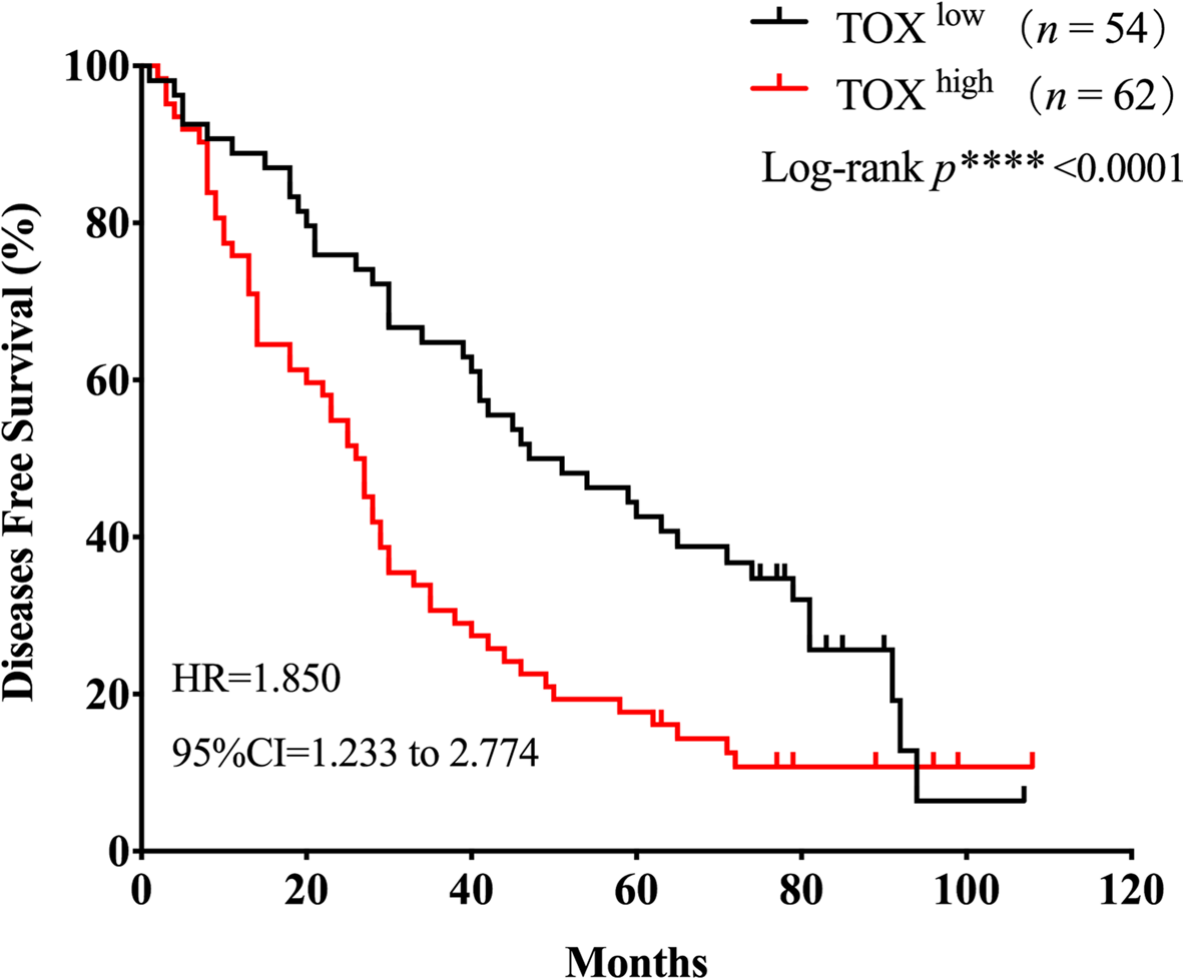
Kaplan-Meier survival curves for disease free survival of patients with ovarian cancer according to the TOX expression scores. Overall survival curves for patients according to the low(*n* = 54) and high(*n* = 62) expression levels of TOX in ovarian cancer tissues (log-rank test and *p* values are shown)

As show in Fig. 5, patients with a shorter DFS (Disease free survival) had higher expression of TOX compared to that with longer DFS (Log-rank p^***^<0.001, Hazard Ratio = 1.850,95% CI = 1.233 to 2.774, Fig. 5). The median DFS of patients with high TOX expression was 26.5 months, whereas that of patients with low TOX expression was 51 months.

Univariate Cox model showed that TOX expression was an independent predictor of short DFS (Hazard Ratio = 1.885, 95%CI = 1.250–2.842, p^**^<0.01). However, multivariate Cox model suggested TOX expression was not an independent predictor (Hazard Ratio = 1.274, 95%CI = 0.791–2.052, *p = 0.319*). Univariate and multivariate analyses showed that T stage and M stage were the independent factors correlated with DFS of ovarian cancer patients (Table 3).

**Table 3:** Univariate and multivariate analyses of the factors correlated with diseases free survival of ovarian cancer patients

Variables	Univariate analysis			Multivariate analysis		
	HR	95%CI	*p* value	HR	95%CI	*p* value
TOX expression	1.885	1.250–2.842	0.002^**^	1.274	0.791–2.052	0.319
Age	1.402	0.937–2.100	0.101			
T stage	4.111	2.455–6.885	< 0.001^***^	3.396	2.007–5.748	< 0.001^***^
N stage	3.130	2.011–4.873	< 0.001^***^	1.095	0.596–2.011	0.771
M stage	4.144	2.559–6.712	< 0.001^***^	2.247	1.177–4.291	0.014^**^
TNM stage	3.052	2.243–4.152	< 0.001^***^			
KI67	1.208	0.806–1.809	0.360			
EGFR	1.037	0.695–1.549	0.858			
PDL-1	1.508	0.964–2.359	0.072			

Statistically significant (p^*^ < 0.05, p^**^ < 0.01, p^***^ < 0.001)

## Discussion

In recent years, TOX has emerged as an important regulator of T cell dysfunction programs in tumors and chronic infections [11–14, 23, 24]. TOX is thought to bind DNA not in a sequence-dependent but structure-dependent manner and facilitate binding of protein cofactors through its transactivation domain [6]. There is a growing evidence that TOX appears to have distinct roles in effector, progenitor exhausted, anergic, terminally exhausted and dysfunctional T cells [25]. TOX expression levels may be pivotal in determining fate and function of T cells. Therefore, some studies have revealed that TOX could predict prognosis in human cancer. Kim et al. showed TOX levels in the tumor infiltrating T cells was found to be highly predictive of OS and anti-PD1 efficacy in melanoma and non-small cell lung cancer patients [10].Han et al. revealed TOX expression levels represented the most terminally exhausted status and tumor antigen reactivity among tumor infiltration T cells in bladder cancer patients [21].Yang et al. investigated the value of TOX expression in predicting prognosis of advanced colorectal cancer [20].They revealed that high TOX expression was negatively correlated with TumorPurity but positively related to ImmuneScore, StromalScore, microsatellite instability status and Consensus Molecular Subtypes 3 typing [20]. In addition, TOX was also found to correlate with prognosis, immune infiltration and T cells exhaustion in lung adenocarcinoma [26]. TOX showed significant impacts on the survival of lung adenocarcinoma patients with early stage, ever smoking or low tumor mutation burden. Furthermore, it was showed that increased TOX expression positively correlated with high immune infiltration levels in most of the immune cell and functional T cells including exhausted T cell in lung adenocarcinoma tissues. That might be the reason why TOX could be a prognosis predicator for lung adenocarcinoma patients [26]. Moreover, TOX correlated not only the prognosis of solid tumor but also hematological malignancy. For example, Liang et al. reported that higher TOX expression was associated with poor OS for patients with acute myeloid leukemia [22]. However, the relationship between TOX expression and prognosis in ovarian cancer has not been comprehensively investigated. Thus, we demonstrated TOX was a potential prognosis-related biomarker in ovarian cancer and suggested a new direction to understand the correlations between TOX, immune infiltration and T cells function in tumor microenvironment.

In this work, we found that TOX expression in ovarian cancer specimens resected from 116 patients by IHC. The results showed TOX was significantly highly expressed in tumor tissues compared with normal adjacent tissues. In addition, association analyses verified that TOX expression was positively correlated with tumor status, Ki67 and PD-L1but not associated with the age of patients and expression of EGFR in tumors. Importantly, we found that high TOX expression predicted poor OS and DFS in patients with ovarian cancer. Univariate and Multivariate Cox analysis confirmed that TOX was an independent predictor of OS in patients with ovarian cancer. Whereas TOX was not an independent predictor of DFS in patients with ovarian cancer. The reason why TOX was not an independent predictor was that TOX expression was significantly associated with tumor stage on which the DFS was tightly related.

## Conclusion

In conclusion, we identified the expression of TOX in 116 patients with ovarian cancer and confirmed that the TOX could independently predicted poor survival and prognosis. Further studies are needed to clarify the underlying mechanism that how TOX can affect the function of T cell and tumor cell in ovarian cancer microenvironment. We hope that detecting TOX expression in ovarian cancer may provide a new biomarker in guiding the treatment strategy for ovarian cancer.

## Materials and methods

### Patients and specimens

The formalin-fixed paraffin-embedded (FFPE) tissues used for study were collected from 116 patients with ovarian cancer who underwent curative surgery from 2010 to 2018. The patients received adjuvant chemotherapy according to their tumor stage. The Disease-free survival (DFS) was defined as the length of time for which patient survived after curative resection without any positive radiological imaging test or death from any cause. Overall survival (OS) was evaluated from the date of surgical resection of the primary tumor to the date of death or the last follow-up. This study was approved by the Ethical Board of the Institutional Review Board of Shanghai Outdo Biotech Company. All the patients provided written informed consent before any study-related procedures.

### Immunohistochemistry (IHC) and immunofluorescence protocol for ovarian cancer

Ovarian cancer tissue microarrays were prepared semi-automatically with the basic protocols include [1] mark donor blocks showing the tissue of interest that corresponds with the H&E-stained slide, and organize for arrays; [2] punch blank recipient blocks of appropriate size for the array (punches with a diameter of 0.6–1 mm); [3] use the arrayer to insert the tissue cores in the recipient arrayer, putting them in the punches of the recipient block according to a predesigned map, under mild vacuum suction and ensuring that the cores lie in the same plane at the cut surface, in accordance with the arrayer manufacturer’s guidelines; [4] cut with meticulous care on a rotary microtome. Then, 5-µm sections from tissue microarrays were baked at 63°C for 1 hour. The sections were then de-paraffinized in xylene, rehydrated using a gradient of ethanol concentrations, boiled in 1 mM Tris-EDTA buffer with a high-pressure cooker (PH-070, Yiheng Company, Shanghai, China) for 3 minutes to retrieve antigens, blocked with 3% hydrogen peroxide for 10 minutes to inhibit activities of endogenous peroxidases and incubated with 10% goat non-immune serum for 20 minutes to reduce non-specific staining. After that, the sections were incubated with rabbit anti-TOX monoclonal antibody (1:500 dilution; ab155768, Abcam, Cambridge, UK), anti-Ki67 monoclonal antibody( 1:200 dilution; ab16667, Abcam, Cambridge, UK), anti-EGFR monoclonal antibody ( 1:200 dilution; ab32077, Abcam, Cambridge, UK) and anti-PD-L1 monoclonal antibody (1:250 dilution; ab213524, Abcam, Cambridge, UK) at 4°C overnight, then incubated with biotin-labeled secondary antibody (Ultrasensitive SP IHC kit, FuZhou MXB Biotechnology, China) at room temperature for 10 minutes, followed by incubation with HRP-conjugated streptavidin (Ultrasensitive SP IHC kit, FuZhou MXB Biotechnology, China) at room temperature for another 10 minutes. Color development was performed with DAB Substrate Kit (Dako, Glostrup, Denmark). Finally, the sections were counterstained with hematoxylin, dehydrated, cleared, and mounted. Multiplexed fluorescent IHC was performed using Opal 7-color Manual IHC Kit (NEL801001KT,PerkinElmer, USA) and VECTASHIELD® HardSet Antifade Mounting Medium (H-1400, Vector Labs, CA,USA) according to the instructions by the manufactures. Cell nuclei were counterstained with 4’,6-Diamidino-2-Phenylindole, Dihydrochloride (DAPI). The multiplexed fluorescence-labelled images were analyzed with automated imaging systems (Vectra Polaris, PerkinElmer, USA; TissueFAXS Spectra, Tissue Gnostics, Austria).

### IHC staining evaluation of TOX, Ki67, EGFR, PD-L1 expressions

Whole tumor slides were randomly reviewed by two independent pathologists based on the intensity and the proportion of positively stained cells. Both reviewers were blinded until two reviews on two separate days were completed. Stain intensity was evaluated with a 4-grading system: 0 = negative, 1 = weak, 2 = moderate and 3 = strong. The percentage of positive cells were scored as follows: 0 for no cell stained, 1 for 1 − 25% of cells stained, 2 for 26–50% of cells stained, 3 for 51–75% of cells stained and 4 for more than 75% of cells stained. Scores for intensity and percentage of positive cells were multiplied. In order to make sure that the stain intensity should be moderate or strong and the percentage of TOX positive cells was larger than 50%, score 8 was determined as the cut off value. Scores < 8 was used to define tumors with low TOX expression and scores ≥ 8 with high TOX expression.

### Statistical analysis

Statistical analyses were performed using SPSS Statistics version 28.0.1 (IBM, Armonk, NY, USA) and Prism version 9.0 (GraphPad, San Diego, CA, USA). The comparison of TOX expression between cancerous tissue and adjacent non-cancerous tissue was tested by Mann-Whitney U test. The correlations between TOX expression and clinical parameters were tested using Fisher’s exact test or Pearson’s *chi*-square test, as appropriate. DFS and OS were evaluated using the Kaplan-Meier method, and log-rank test was used to compare the difference between groups. For the analysis of DFS, data for patients who are alive and had no disease or who were lost to follow-up were censored at the time of the last assessment. For analysis of OS, data for patients who are alive or who were lost to follow-up were censored at the time of the last assessment. All results were considered significant when p^*^ *< 0.05*, p^**^<0.01, p^***^<0.001, p^****^<0.0001.

## Data Availability

The datasets used and/or analyzed during the current study are available from. the corresponding author on reasonable request.

## Abbreviations

HGSC: High-grade serous ovarian cancer
TOX: Thymocyte selection-associated high mobility group box
TME: Tumor microenvironment
OS: Overall survival
DFS: Disease free survival
FFPE: Formalin-fixed paraffin-embedded

## References

[1] Siegel RL, Miller KD, Fuchs HE, Jemal A (2022). Cancer statistics 2022. CA Cancer J Clin.

[2] Lee J-Y, Kim S, Kim YT, Lim MC, Lee B, Jung K-W (2018). Changes in ovarian cancer survival during the 20 years before the era of targeted therapy. BMC Cancer.

[3] Lheureux Stephanie , Gourley C, Vergote I, Oza Amit M (2019). Epithelial ovarian cancer. Lancet.

[4] Lheureux S, Braunstein M, Oza AM (2019). Epithelial ovarian cancer: evolution of management in the era of precision medicine. Cancer J Clin.

[5] Kandalaft LE, Odunsi K, Coukos G (2019). Immunotherapy in Ovarian Cancer: are we there yet?. J Clin Oncol.

[6] O’Flaherty E, Kaye J (2003). TOX defines a conserved subfamily of HMG-box proteins. BMC Genomics.

[7] Wilkinson B, Chen JY, Han P, Rufner KM, Goularte OD, Kaye J (2002). TOX: an HMG box protein implicated in the regulation of thymocyte selection. Nat Immunol.

[8] Aliahmad P, Seksenyan A, Kaye J (2012). The many roles of TOX in the immune system. Curr Opin Immunol.

[9] Seehus CR, Aliahmad P, de la Torre B, Iliev ID, Spurka L, Funari VA (2015). The development of innate lymphoid cells requires TOX-dependent generation of a common innate lymphoid cell progenitor. Nat Immunol.

[10] Kim K, Park S, Park SY, Kim G, Park SM, Cho J-W (2020). Single-cell transcriptome analysis reveals TOX as a promoting factor for T cell exhaustion and a predictor for anti-PD-1 responses in human cancer. Genome Med.

[11] Wang X, He Q, Shen H, Xia A, Tian W, Yu W (2019). TOX promotes the exhaustion of antitumor CD8 + T cells by preventing PD1 degradation in hepatocellular carcinoma. J Hepatol.

[12] Scott AC, Dundar F, Zumbo P, Chandran SS, Klebanoff CA, Shakiba M (2019). TOX is a critical regulator of tumour-specific T cell differentiation. Nature.

[13] Khan O, Giles JR, McDonald S, Manne S, Ngiow SF, Patel KP (2019). TOX transcriptionally and epigenetically programs CD8 + T cell exhaustion. Nature.

[14] Yao C, Sun H-W, Lacey NE, Ji Y, Moseman EA, Shih H-Y (2019). Single-cell RNA-seq reveals TOX as a key regulator of CD8 + T cell persistence in chronic infection. Nat Immunol.

[15] Beltra J-C, Manne S, Abdel-Hakeem MS, Kurachi M, Giles JR, Chen Z (2020). Developmental Relationships of four exhausted CD8 + T cell subsets reveals underlying transcriptional and epigenetic Landscape Control Mechanisms. Immunity.

[16] Bejarano L, Jordāo MJC, Joyce JA (2021). Therapeutic targeting of the Tumor Microenvironment. Cancer Discov.

[17] Ngiow SF, Young A (2020). Re-education of the Tumor Microenvironment with targeted therapies and immunotherapies. Front Immunol.

[18] Binnewies M, Roberts EW, Kersten K, Chan V, Fearon DF, Merad M (2018). Understanding the tumor immune microenvironment (TIME) for effective therapy. Nat Med.

[19] Klemm F, Joyce JA (2015). Microenvironmental regulation of therapeutic response in cancer. Trends Cell Biol.

[20] Yang M, Huang Q, Li C, Jiang Z, Sun J, Wang Z (2021). TOX Acts as a Tumor suppressor by inhibiting mTOR Signaling in Colorectal Cancer. Front Immunol.

[21] Han HS, Jeong S, Kim H, Kim HD, Kim AR, Kwon M (2021). TOX-expressing terminally exhausted tumor-infiltrating CD8(+) T cells are reinvigorated by co-blockade of PD-1 and TIGIT in bladder cancer. Cancer Lett.

[22] Liang C, Zhao Y, Chen C, Huang S, Deng T, Zeng X (2021). Higher TOX genes expression is Associated with Poor overall survival for patients with Acute myeloid leukemia. Front Oncol.

[23] Seo H, Chen J, González-Avalos E, Samaniego-Castruita D, Das A, Wang YH (2019). TOX and TOX2 transcription factors cooperate with NR4A transcription factors to impose CD8 + T cell exhaustion. Proc Natl Acad Sci USA.

[24] Mann TH, Kaech SM (2019). Tick-TOX, it’s time for T cell exhaustion. Nat Immunol.

[25] Philip M, Schietinger A (2022). CD8 + T cell differentiation and dysfunction in cancer. Nat Rev Immunol.

[26] Guo L, Li X, Liu R, Chen Y, Ren C, Du S (2020). TOX correlates with prognosis, immune infiltration, and T cells exhaustion in lung adenocarcinoma. Cancer Med.

